# High-sensitive cardiac troponins in patients undergoing cardiac surgery: friend or foe?

**DOI:** 10.1186/2197-425X-3-S1-A952

**Published:** 2015-10-01

**Authors:** A Wittock, N De Mey, K De Decker, I Brandt, C Van Mieghem, G Cammu, L Foubert

**Affiliations:** OLV Aalst, Anesthesiology and Intensive Care Medicine, Aalst, Belgium; OLV Aalst, Biochemistry, Aalst, Belgium; OLV Aalst, Cardiology, Belgium

## Introduction

High-sensitive cardiac troponin (Hs-cTn) is the new standard cardiac biomarker for the diagnosis of myocardial necrosis [1].

## Objectives

We conducted a prospective study to compare the course and values of the Hs-cTn and CK-MB after CABG and OPCAB surgery. We also evaluated the relationship between values > 10 × 99^th^ percentile URL of CK-MB and Hs-cTn as a possible marker for peri-operative myocardial infarction.

## Methods

After ethics' committee approval and written informed consent all adult patients undergoing elective cardiac surgery between February and November 2014 were included. Exclusion criteria were urgent surgery, GFR < 60 ml/min/1.73m2), CK-MB > 4 Î¼g/L and/or Hs-cTn > 14 ng/L at baseline. Hs-cTn and CK-MB were measured before induction of anaesthesia (baseline), upon arrival in the ICU and at 3, 6, 9, 12, 18, 24 and 48 hrs after arrival. ECGâ?Ts were independently reviewed by two blinded cardiologist. Patients with peri-operative AMI as defined by the third universal definition of AMI (10 × 99th percentile of URL for CK-MB) were excluded for post hoc analysis [2].

## Results

**Figure 1:**
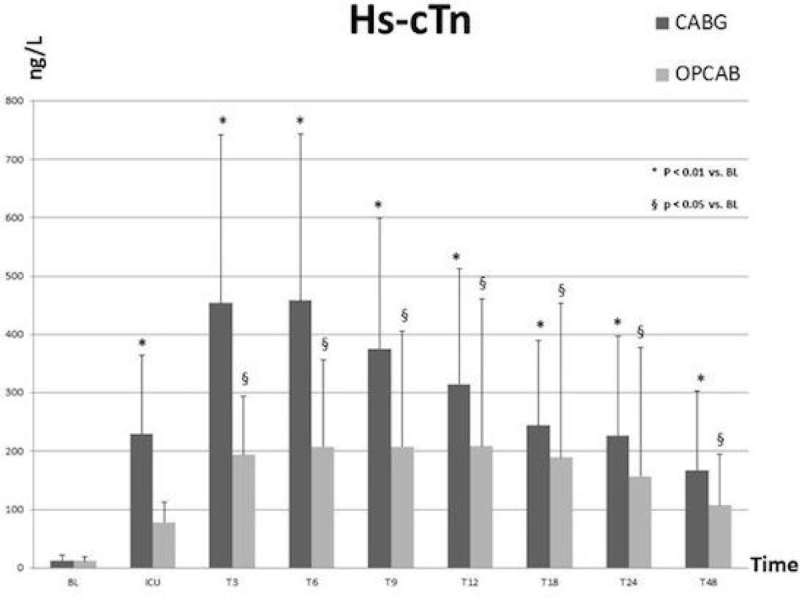
**Hs-cTn in CABG vs OPCAB surgery**.

Of the 121 patients admissible for inclusion, 63 in the CABG group and 21 in the OPCAB group met all inclusion criteria in this preliminary data set. CK-MB values are higher from ICU arrival up to T24 vs. baseline in both CABG and OPCAB (p < 0.0001) with a peak at T3. For Hs-cTn, ICU up to T48 values are higher (p < 0.01) in CABG with a peak at T6, and from T3 to T48 in OPCAB (p < 0.05) vs. baseline (Figure 1). In 62 CABG (98.4 %) and 16 OPCAB patients (76.1 %) all individual Hs-cTn values are above 140 ng/L (= 10 × 99^th^ percentile of URL).

## Conclusions

Both CK-MB and Hs-cTn levels increase significantly after cardiac surgery, and peak later for Hs-cTn than for CK-MB. Values were higher in CABG vs. OPCAB patients for both biomarkers. Postoperative Hs-cTn levels exceed the 10 × 99th percentile of URL in nearly all CABG patients. Our data show an important discrepancy between the 10 × 99th percentile for both biomarkers, and suggest a different definition for post-operative myocardial infarction may be needed when Hs-cTn is used.
